# Primary Pulmonary Epithelial-Myoepithelial Carcinoma: A Case Report and Comprehensive Literature Review of a Rare Lung Neoplasm

**DOI:** 10.7759/cureus.80188

**Published:** 2025-03-07

**Authors:** Sara Morgado, Ana F Santos, Fernando Nogueira

**Affiliations:** 1 Pulmonology Deparment, Unidade Local de Saúde de Lisboa Ocidental, Lisbon, PRT

**Keywords:** case report, endobronchial tumor, multidisciplinary approach, pulmonary epithelial-myoepithelial carcinoma, rare lung tumor

## Abstract

Primary epithelial-myoepithelial carcinoma (EMC) of the lung is a rare subtype of pulmonary tumors. Originating from bronchial submucosal glands, EMC shares histopathological characteristics with its salivary gland counterparts and typically presents as a low-grade malignancy. Due to its rarity, there is limited literature to guide diagnosis and management. We report the case of a 75-year-old male with a history of smoking and chronic conditions who presented with persistent right-sided back pain. Imaging revealed a para-hilar lesion in the right lung, later confirmed as EMC through bronchoscopy and histopathological analysis. Staging studies excluded local invasion and metastasis, and a multidisciplinary team recommended surgical resection. The patient underwent surgery without complications, and histological analysis of the resected specimen confirmed the diagnosis. At follow-up in the pulmonary oncology clinic, no recurrence or metastasis was observed. This case highlights the importance of a multidisciplinary approach for diagnosing and managing rare pulmonary neoplasms. Bronchoscopy and histopathological analysis played a crucial role in achieving an accurate diagnosis. This case contributes to the limited data on EMC and supports surgical intervention as the primary treatment option for localized disease.

## Introduction

Primary epithelial-myoepithelial carcinoma (EMC) of the lung is an exceedingly rare pulmonary neoplasm, accounting for approximately 0.1-0.2% of all lung tumors [1]. These tumors originate from bronchial submucosal glands and share histopathological features with their counterparts in salivary glands. While pulmonary EMCs are generally considered low-grade malignancies, they have the potential for local recurrence and, in rare cases, distant metastasis if not fully excised [2,3].

Diagnosis can be challenging due to the overlap in histological characteristics between EMC and other lung tumors, such as adenocarcinoma and carcinoid tumors. Immunohistochemical profiling plays a pivotal role in distinguishing EMC from these other entities, with markers like CK7, p63, and S-100 being particularly useful [3,4]. Despite the low-grade nature of these tumors, their rarity means there are limited data in the literature to guide diagnostic and therapeutic approaches. Multidisciplinary management involving pulmonology, radiology, pathology, and thoracic surgery is critical to optimizing patient outcomes [5,6].

This case report aims to contribute to the limited understanding of pulmonary EMCs while emphasizing the critical role of a multidisciplinary approach in their diagnosis and management.

## Case presentation

A 75-year-old male, with an Eastern Cooperative Oncology Group (ECOG) performance status of 1 and a retired welder, with a smoking history of 20 pack-years, hypertension, type 2 diabetes mellitus, and dyslipidemia, presented to his primary care physician with persistent right-sided back pain exacerbated by movement. He denied any other associated symptoms, including cough, hemoptysis, or weight loss. 

**Figure 1:**
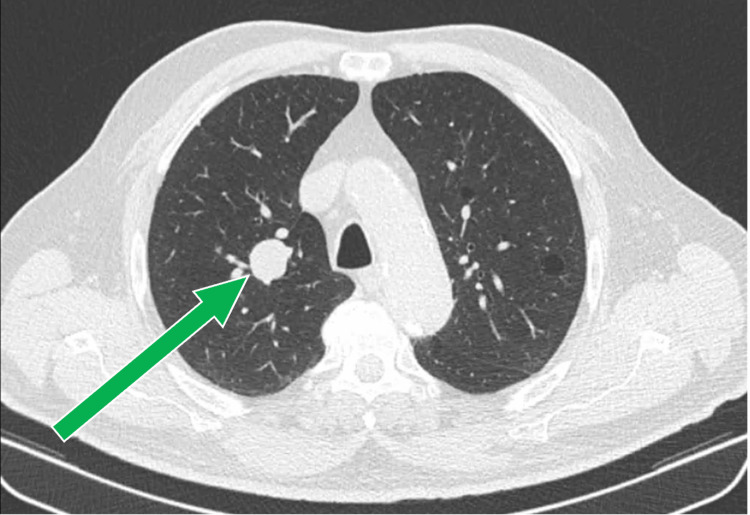
Chest CT - axial plane. The green arrow indicates the right para-hilar lesion.

**Figure 2:**
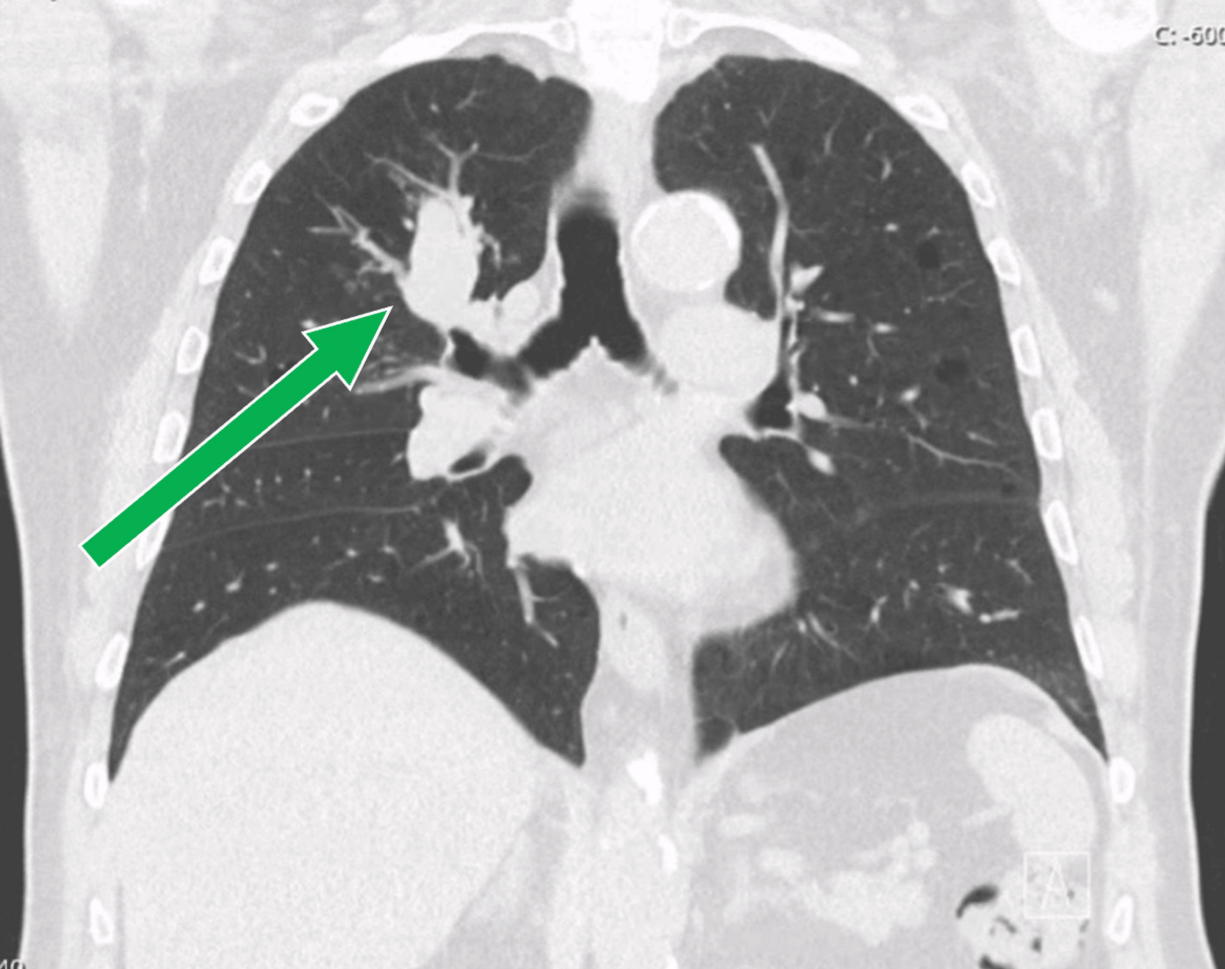
Chest CT - coronal plane. The green arrow indicates the right para-hilar lesion.

A chest computed tomography (CT) scan revealed a 3.6 x 2.0 cm right para-hilar lesion with smooth borders, raising suspicion of an atypical lesion (Figures 1, 2). The patient was subsequently referred to a pulmonology consultation for further evaluation. On clinical examination, he was eupneic in room air with normal peripheral oxygen saturation. Lung auscultation revealed diminished vesicular breath sounds globally, without adventitious sounds.

**Figure 3:**
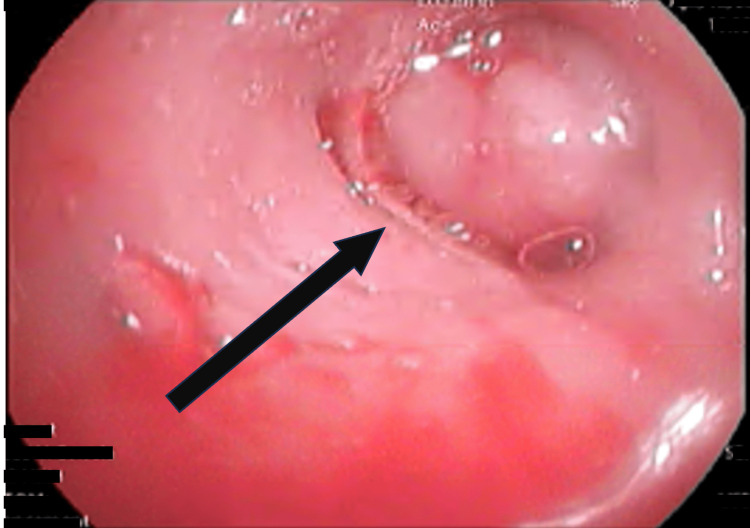
Bronchoscopy: endobronchial mass in the right upper lobe bronchus (black arrow).

**Figure 4:**
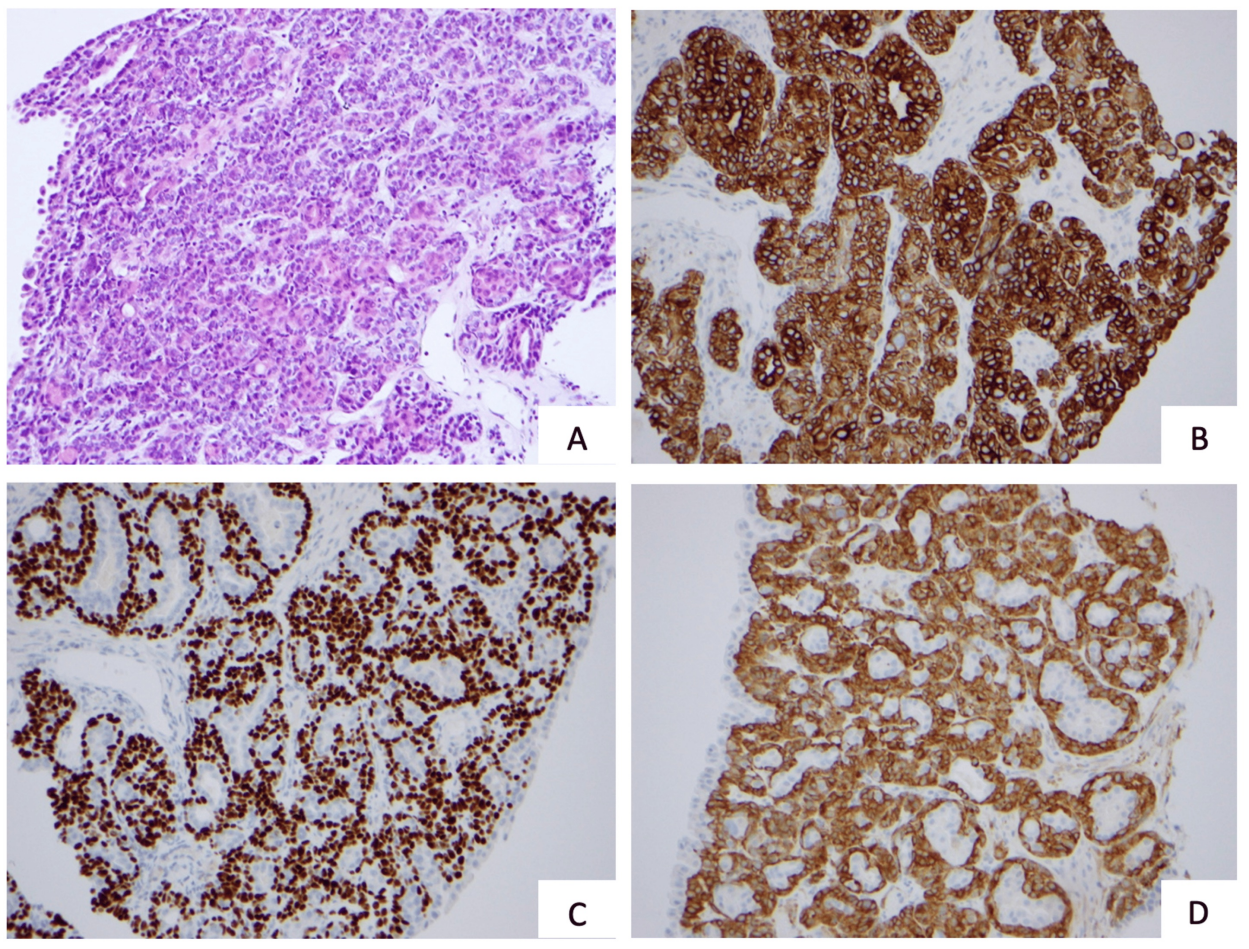
Histopathological examination of bronchial biopsy. (A) Hematoxylin and eosin 200x. (B) CK7 200x. (C) p63 200x. (D) Calponin 200x.

Bronchoscopic assessment identified an endobronchial mass in the right upper lobe bronchus (B1), causing obstruction (Figure 3). Cytological analysis of bronchial brushings and secretions suggested neoplastic cells. The biopsy revealed bronchial mucosa with a chorion occupied by a biphasic salivary-type tumor with focal myxoid stroma. Immunohistochemical analysis demonstrated CK7 positivity in glandular cells, as well as p63, calponin, and S100 positivity in basal and myoepithelial cells, with TTF1 negativity; Ki67 was expressed in approximately 15% of the cells. These findings support the diagnosis of EMC (Figure 4).

For staging purposes, a positron emission tomography-computed tomography (PET-CT) scan demonstrated abnormal hypermetabolism (SUVmax 3.6) in the para-hilar lesion of the right upper lobe, measuring 4.0 x 2.0 cm, without evidence of hypermetabolic activity elsewhere suggestive of malignancy. A cranial CT scan with contrast showed no evidence of brain metastasis. In addition, a neck CT scan was performed to exclude a primary salivary gland tumor, and no primary lesion was identified in the salivary glands, ruling out metastatic disease from an extrathoracic site.

The case was discussed in a multidisciplinary oncologic pulmonology board, which concluded that surgical resection was the most appropriate treatment due to the tumor's low-grade malignancy and the absence of metastasis. The patient underwent thoracic surgery without complications, and histopathological examination of the resected specimen confirmed the diagnosis of primary pulmonary EMC with clear surgical margins.

The patient was referred to the pulmonary oncology clinic for postoperative follow-up. To date, over 12 months of monitoring, no evidence of recurrence or metastasis has been observed. Regular follow-up remains ongoing to manage the low but present risk of recurrence.

## Discussion

Primary pulmonary EMC is an exceedingly rare entity, posing significant diagnostic and therapeutic challenges. Its rarity, combined with histological and immunohistochemical similarities to other primary lung tumors and salivary gland neoplasms, necessitates a thorough diagnostic workup. Pulmonary EMC originates from the submucosal glands of the bronchial tree and shares histopathological features with salivary gland EMCs, highlighting the importance of ruling out extrathoracic primary sites during the diagnostic process [1,3].

In this case, the patient presented with nonspecific symptoms, such as back pain, which led to the incidental discovery of the pulmonary lesion. This underscores the importance of maintaining a high index of suspicion for underlying malignancy in patients with persistent symptoms and risk factors, such as a history of smoking. Imaging studies, particularly computed tomography (CT) and positron emission tomography-computed tomography (PET-CT), were crucial in characterizing the lesion and excluding regional or distant metastatic disease. PET-CT findings of localized hypermetabolism, in conjunction with the absence of hypermetabolic lymph nodes or distant lesions, strongly suggested a primary tumor with no evidence of dissemination, aligning with the literature on the typically localized nature of pulmonary EMC [2,3].

Bronchoscopy, a cornerstone in the evaluation of endobronchial lesions, provided direct visualization of the tumor and facilitated tissue sampling. Histopathological examination revealed the biphasic architecture characteristic of EMC, with epithelial and myoepithelial components, while immunohistochemistry played a decisive role in confirming the diagnosis. Positive staining for CK7, p63, and partial expression of S-100 distinguished this tumor from other pulmonary neoplasms, such as adenocarcinoma, carcinoid tumors, and adenoid cystic carcinoma, as emphasized in previous studies [4,5]. This highlights the essential role of advanced pathological techniques in the accurate diagnosis of rare tumors.

Surgical resection remains the gold standard for the treatment of localized pulmonary EMCs. In this case, a multidisciplinary discussion concluded that surgery was the optimal approach, given the tumor’s low-grade nature and absence of metastasis. Complete resection with clear margins was achieved, and the histopathological analysis of the resected specimen confirmed the diagnosis. This aligns with findings from Chen et al. (2022) and Sharma et al. (2022), who reported that surgical excision is associated with excellent outcomes and minimal recurrence risk for low-grade pulmonary EMC [1,6].

While adjuvant therapies, including chemotherapy and radiotherapy, are generally reserved for high-grade or incompletely resected EMCs, their role in low-grade cases like this one remains limited. The tumor's low mitotic index and indolent behavior further support the preference for surgical management over systemic therapies [6,7]. However, long-term follow-up is crucial, as there remains a small but present risk of local recurrence or, less commonly, distant metastasis. França et al. (2021) noted that although EMC is typically indolent, recurrences have been reported, underscoring the importance of vigilant monitoring [4].

This case highlights several key considerations in the management of pulmonary EMC. First, it reinforces the necessity of a systematic diagnostic approach that integrates imaging, bronchoscopy, histopathology, and immunohistochemistry to achieve an accurate diagnosis. Second, it emphasizes the value of a multidisciplinary team in guiding treatment decisions, particularly for rare tumors where evidence-based guidelines are limited. Lastly, it demonstrates that surgical resection offers favorable outcomes in localized disease, with the potential for long-term disease-free survival.

Given the limited number of reported cases, this case contributes to the growing body of literature on pulmonary EMC and underlines the need for further documentation and research. Future studies are essential to better understand the biological behavior, recurrence risk, and long-term prognosis of this rare tumor, as well as to explore the role of adjuvant therapies in more aggressive or advanced cases.

## Conclusions

This case report describes a rare diagnosis of primary pulmonary EMC, a low-grade malignancy with distinct histopathological characteristics. Diagnosis required a combination of imaging, bronchoscopy, and immunohistochemistry, highlighting the value of a multidisciplinary approach. Surgical resection was effective for managing localized EMC, with favorable outcomes and no evidence of recurrence or metastasis at 12 months follow-up. Documenting such cases is vital to improve understanding and guide future diagnosis and treatment of this rare tumor.
